# Genomic comparison of *Clostridium* species with the potential of utilizing red algal biomass for biobutanol production

**DOI:** 10.1186/s13068-018-1044-9

**Published:** 2018-02-15

**Authors:** Chongran Sun, Shuangfei Zhang, Fengxue Xin, Sabarathinam Shanmugam, Yi-Rui Wu

**Affiliations:** 1 Department of Biology, Shantou University, Shantou, 515063 Guangdong China; 2 Guangdong Provincial Key Laboratory of Marine Biotechnology, Shantou University, Shantou, 515063 Guangdong China; 3 STU-UNIVPM Joint Algal Research Center, Shantou University, Shantou, 515063 Guangdong China; 4 State Key Laboratory of Materials-Oriented Chemical Engineering, College of Biotechnology and Pharmaceutical Engineering, Nanjing Tech University, Nanjing, 211816 Jiangsu China

**Keywords:** *Clostridium* species, Genome evolution, Metabolic pathways, Biofuels, Algae

## Abstract

**Background:**

Sustainable biofuels, which are widely considered as an attractive alternative to fossil fuels, can be generated by utilizing various biomass from the environment. Marine biomass, such as red algal biomass, is regarded as one potential renewable substrate source for biofuels conversion due to its abundance of fermentable sugars (e.g., galactose). Previous studies focused on the enhancement of biofuels production from different *Clostridium* species; however, there has been limited investigation into their metabolic pathways, especially on the conversion of biofuels from galactose, via whole genomic comparison and evolutionary analysis.

**Results:**

Two galactose-utilizing Clostridial strains were examined and identified as *Clostridium acetobutylicum* strain WA and *C. beijerinckii* strain WB. Via the genomic sequencing of both strains, the comparison of the whole genome together with the relevant protein prediction of 33 other *Clostridium* species was established to reveal a clear genome profile based upon various genomic features. Among them, five representative strains, including *C. beijerinckii* NCIMB14988, *C. diolis* DSM 15410, *C. pasteurianum* BC1, strain WA and WB, were further discussed to demonstrate the main differences among their respective metabolic pathways, especially in their carbohydrate metabolism. The metabolic pathways involved in the generation of biofuels and other potential products (e.g., riboflavin) were also reconstructed based on the utilization of marine biomass. Finally, a batch fermentation process was performed to verify the fermentative products from strains WA and WB using 60 g/L of galactose, which is the main hydrolysate from algal biomass. It was observed that strain WA and WB could produce up to 16.98 and 12.47 g/L of biobutanol, together with 21,560 and 10,140 mL/L biohydrogen, respectively.

**Conclusions:**

The determination of the production of various biofuels by both strains WA and WB and their genomic comparisons with other typical *Clostridium* species on the analysis of various metabolic pathways was presented. Through the identification of their metabolic pathways, which are involved in the conversion of galactose into various potential products, such as biobutanol, the obtained results extend the current insight into the potential capability of utilizing marine red algal biomass and provide a systematic investigation into the relationship between this genus and the generation of sustainable bioenergy.

**Electronic supplementary material:**

The online version of this article (10.1186/s13068-018-1044-9) contains supplementary material, which is available to authorized users.

## Background

Increasing concerns about greenhouse gas-mediated climate change and the current high energy demands are driving the development of renewable and sustainable sources that can replace non-renewable fossil fuels [1–3]. As one of the most promising renewable fuels, biofuels can be generated via the microbial fermentation process using various biomass from the environment [4, 5]. Biobutanol, bioethanol and biohydrogen are crucial biofuels that are regarded as major optional substitutes for fossil fuels [6–8], and the development of this sustainable and renewable biomass should facilitate and advance of biofuels production.

Marine biomass, which has gradually attracted attentions, is considered to be one of the potential sources for biofuels conversion due to the high amount of carbohydrates [9–11]. Seaweeds represent an abundant and renewable biomass with fast-growing characteristics that are beneficial for the production of third generation biofuels [12, 13], which are often referred as marine macro-algae including red, brown and green algae [14]. As one of predominant sources of marine biomass, red algal biomass is comprised of agar and cellulose that can be hydrolyzed into various simple sugars (e.g., glucose or galactose) for biofuels fermentation [15–17] and it has been utilized as a sustainable and environmentally friendly feedstock for biohydrogen production over the past few decades [18]. Furthermore, there is no lignin found within most red algal biomass, which further reduces the cost of the pretreatment to generate utilizable substrates for microorganisms to produce biofuels [19].

Microorganisms from the *Clostridium* genus are diverse and include a large group of anaerobic, endospore-forming bacteria, which possesses hundreds of species [20], and the majority are recognized as the most notable native cellular factories due to their vast range of substrates utilization and metabolic diversity for the generation of various bio-products [21, 22]. Therefore, one of the most feasible options is the application of Clostridial strains to produce biobutanol by utilizing algal biomass through the acetone–butanol–ethanol (ABE) fermentation process [23]. *C. pasteurianum* was first reported to be capable of converting algal biomass into limited butanol (0.13 g/L) with the presence of 4% of glycerol [24]. In recent studies, Ellis et al. [25] adopted *C. saccharoperbutylacetonicum* to utilize wastewater algal biomass to produce butanol with the addition of xylanases and cellulases, whereas green seaweed was also used for butanol production by strains *C. acetobutylicum* and *C. beijerinckii* with the co-metabolism of glucose and xylose [26]. Potts et al. [27] further processed 15.2 g/L of reducing sugar recovered from macro-algae to enhance butanol production up to 4 g/L using both strains of *C. beijerinckii* and *C. saccharoperbutylacetonicum.* Moreover, other solventogenic Clostridial strains, such as *C. tetanomorphum* ATCC 49273, *C. aurantibutyricum* NCIMB 10659 and *C. beijerinckii* NCIMB 8052, were also reported to obtain biobutanol from another marine macroalgae feedstock, namely, *Ceylon moss* [28].

In addition to biobutanol, other bio-products can also be obtained via Clostridial fermentation from algal biomass. Park et al. [14] employed anaerobic sewage sludge microflora for biohydrogen production using macro-algae biomass (*Laminaria japonica*), and wastewater algal biomass was used to ferment bioethanol via the cellulolytic strain *C. phytofermentans* DSM 1183 [29]. *Clostridium* species was also reported to co-produce butanol with riboflavin (vitamin B_2_), a yellow water-soluble vitamin used as an important cofactor in cells, which also provides an economically practicable way to further exploit the process using algal biomass [30]. In addition, the production of butyric acid using red algae *Gelidium amansii* as the carbon source was also presented [31]. Sivagurunathan et al. [32] applied the combined inoculation strategy to improve biohydrogen production from galactose, which is the main hydrolysate from algal biomass, and Sund et al. [33] evaluated the different roles of *C. acetobutylicum* in the galactose utilization pathway. Therefore, the Clostridial strains, especially *C. acetobutylicum* and *C. beijerinckii*, possess the potential to generate value-added bio-products by using galactose, algal hydrolysate or even algal biomass as substrates.

Although there are large amounts of natural microbial isolates with various metabolic pathways involved in the utilization of biomass and the conversion of biofuels [34], the lack of well-developed genetic tools and the complicated physiological characteristics from various microbial strains resulted in a limited understanding and development of certain microbial groups [4], and the comprehensive comparison of the respective strains and their specific capabilities are still lacking [21]. With further investigation via genetic and genomic analysis and the recent efforts for the metabolically engineered Clostridial strains, their innate capabilities, especially the possible potential metabolites and the utilization of recalcitrant substrates, can be demonstrated [22, 34]. In addition, the phylogeny function based methodology that was well known for the study of genomic libraries [35], special functional enzymes [36] and ecosystem analysis [37] can also be established to investigate the relationship between their phenotypes and genotypes. Therefore, an approach dealing with the phylogenetic tree based on whole genomic sequences and a functional comparison on genomic scale could also be applied to analyze the metabolic pathway involved in the generation of biofuels or bio-products by *Clostridium* species.

In this study, two newly isolated galactose-utilizing *Clostridium* strains were identified as *C. acetobutylicum* strain WA and *C. beijerinckii* strain WB via the whole genomic sequencing. In addition to make comparisons of the genome profiles based upon the genomic features of the other 33 Clostridial strains, three representative strains, including *C. beijerinckii* NCIMB 14988, *C. diolis* DSM 15410 and *C. pasteurianum* BC1, were selected to reveal the critical differences among their respective metabolic potential in utilizing algal biomass for various biofuels and/or biochemicals production by comparison with strains WA and WB, which was further experimentally verified via the ABE fermentation process. In total, this work not only presents the metabolic pathway of the bioconversion of galactose to biobutanol by *Clostridium* sp. strain WA and WB, but also builds up a comprehensive investigation on the metabolic potential of other industrial bio-products using Clostridial strains and algal biomass through whole genomic comparison and evolutional analysis.

## Results and discussion

### Genomic features of strains WA and WB

Through genomic sequencing and annotation, the metabolic pathways of biofuels/biochemicals production by the galactose-utilizing strains, *Clostridium* sp. WA and WB, were analyzed and compared to achieve better insights. It was observed from Table 1 that the genome of strain WA is comprised of a circular chromosome and a mega-plasmid, consisting of a genomic size of 4.07 Mbp with a G+C content of 30.8%. However, the genome of strain WB displayed a much larger genome of 5.78 Mbp with a similar G+C content of 29.7% and there was no plasmid detected during the assembly of strain WB. The final annotation of strain WA and WB resulted in 3878 and 5085 coding sequences (CDSs), respectively. A total of 104 RNA sequences, including 31 rRNA genes (5S, 16S and 23S) and 72 tRNA genes, were found in strain WA; however, strain WB appeared to have less rRNA or tRNA genes detected within its genome.

**Table 1:** General genome features of strains WA and WB

Bacterial strains	*C. acetobutylicum* WA	*C. beijerinckii* WB
Genome size (Mbp)	4.07	5.78
Contigs	1	1
G+C%	30.8	29.7
Genes	3927	5142
CDSs	3878	5085
tRNAs	72	56
rRNAs	31	4
Plasmid DNA	1	0

### Whole genome-based phylogenetic analysis of strains WA and WB

**Fig. 1:**
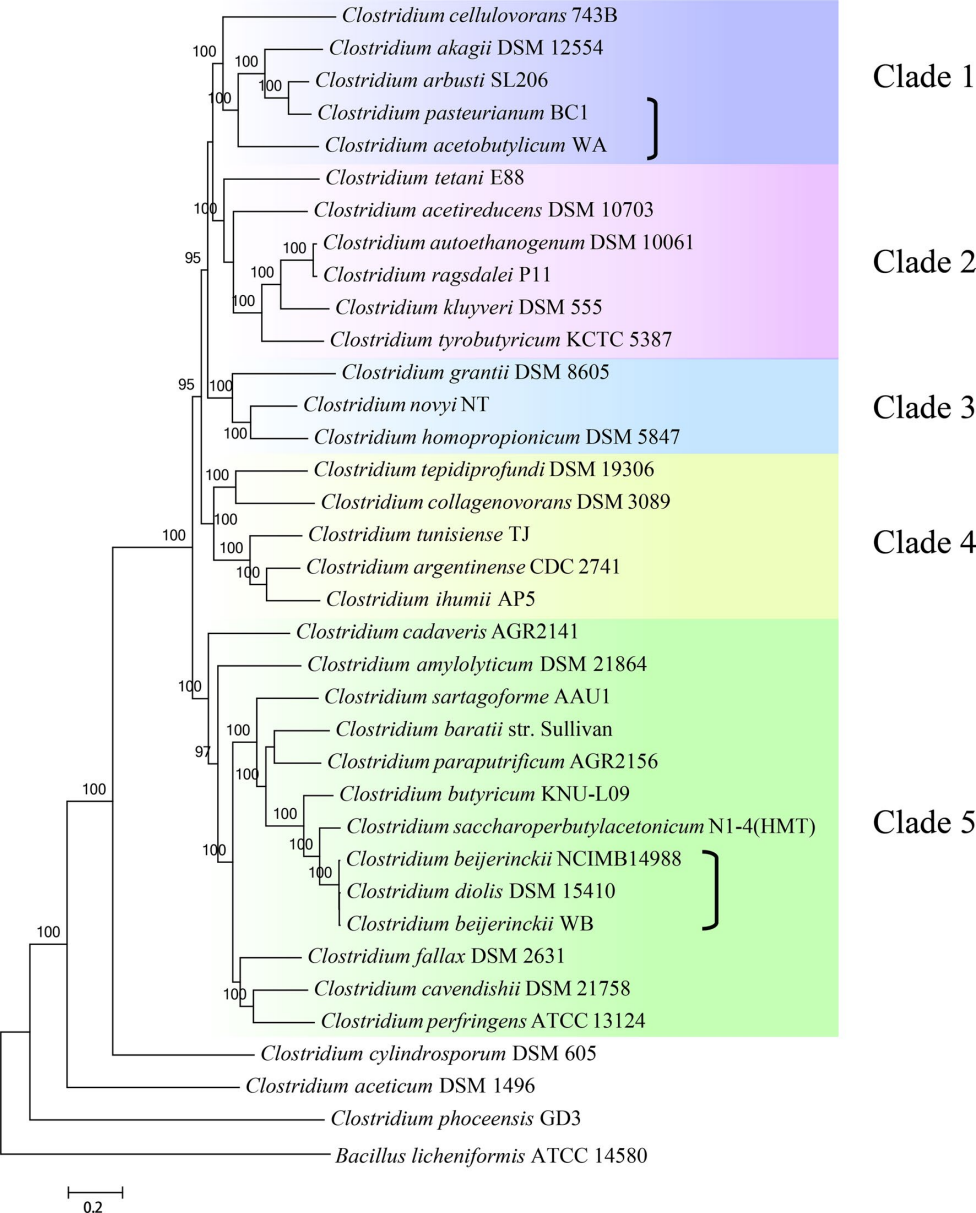
Construction of the whole-genome phylogenetic tree of 35 Clostridial strains based on the RAxML maximum likelihood methodology. The values close to each internal branch indicate the posterior probability, and those lower than 90 are not shown

By a composition-heterogeneous model in the P4 software package, a randomized axelerated maximum likelihood (RAxML) phylogenetic tree [38] was constructed based on the whole genomes from strains WA and WB (Additional file 1: Tables S1 and S2) together with 33 other available Clostridial strains according to the concatenated alignment of 129 bacterial single copy marker genes with a total of 10793 amino acid sites, and *Bacillus licheniformis* ATCC 14580 was set as an outgroup. It was observed that these strains were phylogenetically placed into five clades (Fig. 1). The genomes from strains *C. acetobutylicum* WA, *C. arbusti* SL206, *C. akagii* DSM 12554, *C. cellulovorans* 743B and *C. pasteurianum* BC1 formed a robustly monophyletic group with the same evolutionary clade (Clade 1), whereas strains *C. beijerinckii* NCIMB 14988, *C. diolis* DSM 15410 and *C. beijerinckii* WB were clustered together into another single subclade (Clade 5). These two newly sequenced strains (strains WA and WB) reveal a relatively far evolutionary relationship/distance, which indicates the possible distinction in their metabolism related to biomass utilization and the conversion of bio-products.

**Fig. 2:**
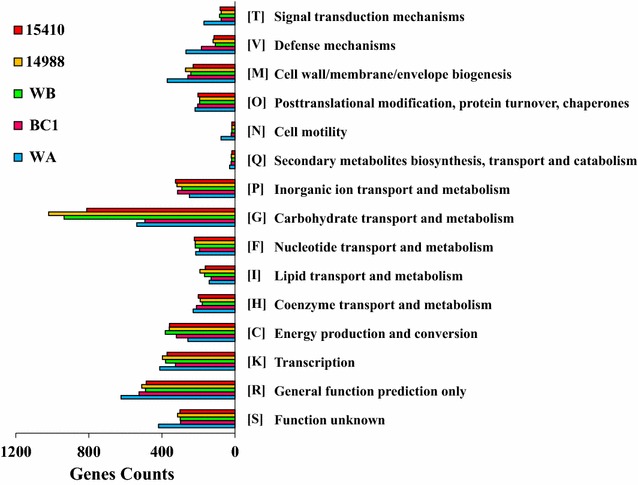
The distribution of genes involved in various metabolism within the genomes of five representative Clostridial strains

*Clostridium* strains with the potential of utilizing various biomass (e.g., corn cobs, cassava and rice bran) and the production of biofuels (e.g., butanol) were mainly classified within Clade 1 and Clade 5 such as *C. cellulovorans* 743B and *C. saccharoperbutylacetonicum* N1-4 [39–41]. Genomes from the same clustered group usually appear to have similar metabolic functions, which demonstrates a comprehensive way to understand certain isolated strain based on the available reference genomes from other strains. Therefore, according to the phylogenetic location and genomic characteristics, the reference genomes from three other Clostridial strains, including *C. beijerinckii* NCIMB14988, *C. diolis* DSM 15410 and *C. pasteurianum* BC1, together with the genomes of strains WA and WB, were selected to determine the differences from their metabolic pathways and provide directions for future fermentation. Via the classification from annotated genes using the COG database (Fig. 2), the distributions of the functional proteins were observed to be the same trend for the affiliation of the selected strains (Fig. 1). The result also suggests that the phylogenetic analysis based on single-copy gene families could be utilized to cluster the individual microbial strains from the same genera into the specific clade using the interaction of the functional genes, which provides more insights rather than using only 16S rRNA gene sequences [42].

### Determination of the genomic characteristics from diverse Clostridial strains

**Fig. 3:**
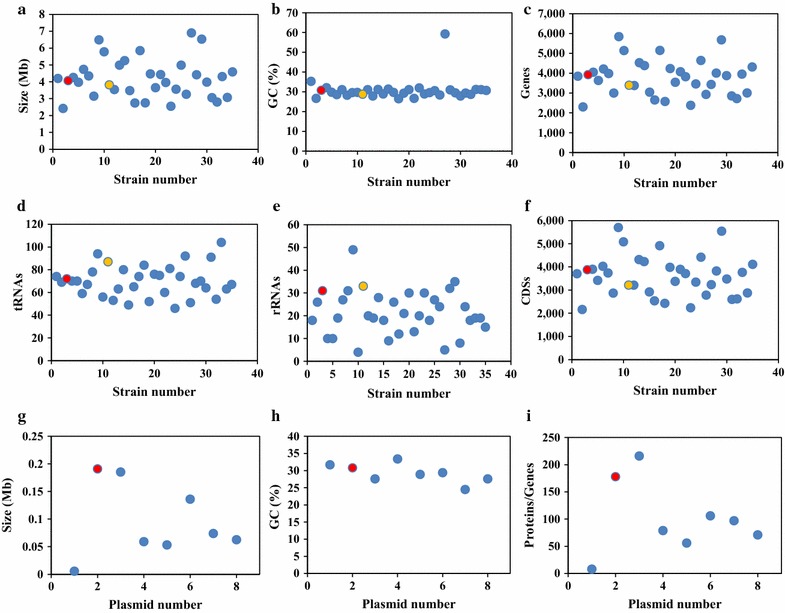
Comparison of the genomic features of 35 Clostridial strains. **a**–**f** Characteristics of the chromosomal DNA from 35 strains (Additional file 1: Table S1); **g**–**i** characteristics of the plasmid DNA from 8 strains (Additional file 1: Table S2). The red color indicates chromosomal or plasmid DNA from strain WA, and the yellow color represents chromosomal DNA from strain WB

To better understand the basic divergence among reference strains, the genomic features of 35 total Clostridial strains were analyzed and assigned to a relatively clear genome classification (Fig. 3), and it was observed that different species, even from the same genus of *Clostridium*, possess the significant divergences such as ~ 2.5–7.0 Mb range for the genome size, ~ 2000–6000 range for the genes/proteins and ~ 50–100 range for the tRNA. This possibly led to the distinction on their respective metabolism. The genome of strain WA was observed to be within the median level from those analyzed genomes, whereas strain WB showed a relatively larger genome with more genes/CDSs but less RNAs from its own genome. In addition to the chromosomal DNA, 8 out of 35 strains were determined to have their own separate circular plasmid. The mega-plasmid of WA (pWA), which encoded a total of 178 proteins with the sol operon, is similar to the reported mega-plasmid (pSOL1) from *C. acetobutylicum* ATCC 824 that contained one vital gene, namely, aldehyde/alcohol dehydrogenase (*aad*) involved in biobutanol generation [43]. *C. saccharoperbutylacetonicum* N1-4(HMT), which is another biosolvents-producing strain, carried a similar size of mega-plasmid (Csp_135p, 0.136 Mbp) that is apparently not related to the formation of solvents. However, those smaller plasmids (50–750 kbp) from *C. aceticum* DSM 1496, *C. kluyveri* DSM 555, *C. pasteurianum* BC1, *C. tetani* E88 and *C. tyrobutyricum* KCTC 5387 were found without a known role in the Clostridial physiological process [44].

### Comparison of the genome-wide metabolic pathway from representative Clostridial strains

The result obtained from the alignment using the COG database indicated that the distinction of gene counts among the five strains involved the pathways in carbohydrate transport and metabolism (Fig. 2). Both strains WA and BC1 have more genes participating in various metabolic processes; however, their genes referring to the carbohydrate transport and metabolism were found to be much less than that from the other three strains. *Clostridium* species exhibit a broad substrate range but there have been limited studies of the mechanisms involved in regulation of uptake and metabolism of fermentable carbohydrates [45]. The presence of numerous phosphotransferase systems (PTS) is reported to be significantly related to the uptake of sugars [46]. For example, *C. acetobutylicum* utilized phosphotransferase system (PTS) transporters for the uptake of disaccharides and hexoses, whereas pentoses were primarily taken up by ATP-binding cassette (ABC) transporters [47]. However, apart from common PTS systems (e.g., PTS-Glc-crr, glucose-specific PTS) involved in both strains WA and WB, more PTS systems were exclusively present in the metabolism of strain WB, such as PTS-Dga-dgaB/dgaC/dgaD (d-glucosaminate specific PTS), PTS-Gam-agaC (galactosamine-specific PTS) and PTS-Ula-ulaA/sgaT (ascorbate-specific PTS), which contributed to the higher number of genes that participated in carbohydrate transport and metabolism in strain WB.

**Fig. 4:**
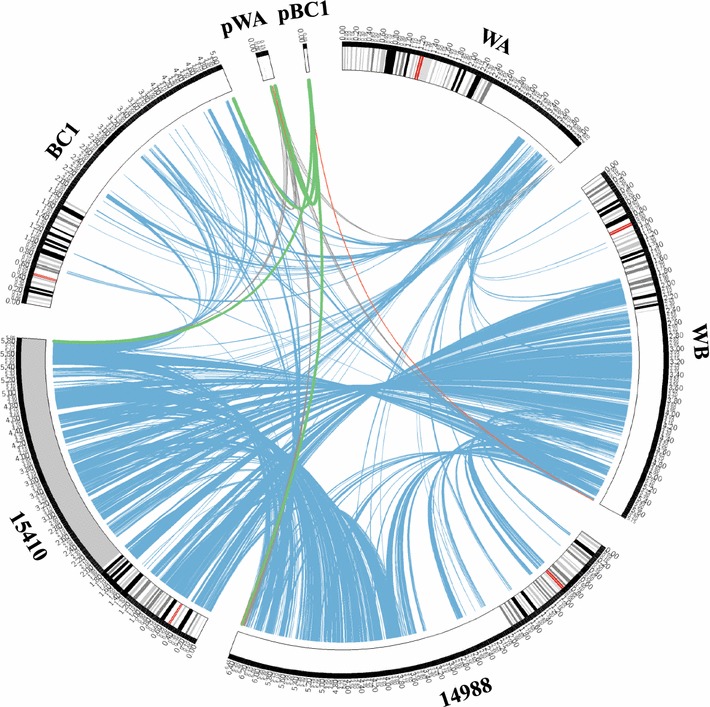
The co-assembly map of the whole genomes from five representative Clostridial strains via Circos analysis. WA: strain WA; WB: strain WB; 14988: *C. beijerinckii* NCIMB 14988; 15410: *C. diolis* DSM 15410; BC1: *C. pasteurianum* BC1; pWA: plasmid of strain WA; and pBC1: plasmid of *C. pasteurianum* BC1

When processing genomic comparison and metabolic reconstruction, analysis of the metabolic capabilities of different Clostridial strains is necessary to consider the relationship between microorganisms and substrates to understand their requirements of carbon catch and energy delivery [4]. The entire genomic distinction among the above five selected Clostridial strains together with two plasmids were compared, and the locations of those obvious syntenic blocks are both highlighted within a circle co-assembly map (Fig. 4). It is worth mentioning that most regions of strain DSM 15410 can match to partial genomic regions of strain WB and NCIMB 14988. However, few similar regions were found from strains WA and BC1 when comparing with the other three strains and even when supplementing their plasmids into the whole genomes. Similarly, few of the same regions could be detected between the galactose-utilizing strain WA and WB (Additional file 2: Figure S1), and most of the genes were involved in maintaining the basic metabolic process such as glycolysis (galactose consumption), TCA cycle and butanoate metabolism (butanol generation). As judged from the genome repertoire (Table 2), five Clostridial strains were able to utilize various saccharides, such as mannose, fructose, glucose and cellulose, to produce various bio-products, and genes from the plasmids in both strains WA and BC1 were also found to participate in the metabolic pathways involved in biofuels generation, especially those from strain WA. However, there are still many differences observed with each other in a metabolism series, such as carbohydrate and energy metabolism.

**Table 2:** Comparison of the crucial metabolic pathways among the five representative Clostridial strains

Functional pathways (meta pathways labels)	Clostridial strains				
	WA	BC1	WB	DSM 15410	NCIMB 14988
Carbohydrate metabolism					
Beta-1,4-d-mannosyl-*N*-acetyl-d-glucosamine degradation (PWY-7586)			Y		
Cellulose biosynthesis (PWY-1001)	Y	Y			
Cellulose degradation II (PWY-6788)	Y	Y	Y	Y	Y
d-Galactose degradation V (Leloir pathway, PWY66-422)	Y	Y	Y	Y	
d-Galacturonate degradation I (GALACTUROCAT-PWY)	Y	Y			
Ethanol degradation IV (PWY66-162)		Y	Y	Y	Y
Fucose degradation (FUCCAT-PWY)					Y
GDP-mannose biosynthesis (PWY-5659)			Y		
Gellan degradation (PWY-6827)	Y				
Gluconeogenesis I (GLUCONEO-PWY)			Y	Y	Y
Glycerol degradation I (PWY-4261)	Y	Y	Y	Y	Y
Glycogen biosynthesis I (GLYCOGENSYNTH-PWY)	Y	Y			
Glycolysis I (GLYCOLYSIS)	Y	Y	Y	Y	Y
Glycolysis III (ANAGLYCOLYSIS-PWY)			Y	Y	Y
Lactose degradation III (BGALACT-PWY)	Y				
l-Ascorbate degradation II (PWY-6961)			Y		
l-Rhamnose degradation I (RHAMCAT-PWY)					Y
Maltose degradation (MALTOSECAT-PWY)	Y	Y			
Pentose phosphate pathway (NONOXIPENT-PWY)	Y	Y			
Pyruvate fermentation to acetone (PWY-6588)	Y	Y	Y	Y	Y
Pyruvate fermentation to butanoate (CENTFERM-PWY)	Y				
Sucrose biosynthesis II (PWY-7238)	Y	Y	Y	Y	Y
Sucrose degradation III (PWY-621)	Y	Y			
TCA cycle VIII (REDCITCYC)			Y	Y	Y
Trehalose degradation I (TREDEGLOW-PWY)	Y				
UDP-d-galactose biosynthesis (PWY-7344)	Y	Y	Y	Y	Y
UDP-glucose biosynthesis (PWY-7343)	Y	Y	Y	Y	Y
Energy metabolism					
[2Fe-2S] iron-sulfur cluster biosynthesis (PWY-7250)	Y	Y	Y	Y	Y
Hydrogen production (PWY-6759)	Y	Y	Y	Y	Y
Hydrogen to dimethyl sulfoxide electron transfer (PWY0-1577)	Y	Y	Y	Y	Y
Phosphatidylethanolamine biosynthesis I (PWY-5669)	Y		Y	Y	Y
Sulfoquinovosyl diacylglycerol biosynthesis (PWYQT-4427)	Y				
Nitrogen fixation I (N2FIX-PWY)	Y	Y	Y	Y	
Superoxide radicals degradation (DETOX1-PWY)	Y				
Thioredoxin pathway (THIOREDOX-PWY)	Y				
Urate biosynthesis/inosine 5-phosphate degradation (PWY-5695)			Y		
Metabolism of cofactors, vitamins and others					
Acetate conversion to acetyl-CoA (PWY0-1313)		Y			
Acetate formation from acetyl-CoA I (PWY0-1312)	Y	Y	Y	Y	Y
Acyl-CoA hydrolysis (PWY-5148)	Y				
Phosphopantothenate biosynthesis I (PANTO-PWY)	Y	Y	Y	Y	Y
Riboflavin metabolism (RIBOFLAVIN-PWY)	Y	Y	Y	Y	Y
Fatty acid biosynthesis initiation I (PWY-4381)	Y	Y			
CDP-diacylglycerol biosynthesis III (PWY-5981)			Y	Y	Y
Poly-hydroxy fatty acids biosynthesis (PWY-6710)	Y	Y	Y	Y	Y
2,3-Dihydroxybenzoate biosynthesis (PWY-5901)	Y	Y			

“Y” indicates the presence of relevant metabolic pathway

As determined from Fig. 2, the overall trend of gene distribution in the five strains was similar. Although the number of genes is not directly proportional to the total number of metabolic pathways, there are obvious differences found from the pathway of carbohydrate transport and metabolism. For example, the quantity of genes in strain WB, DSM 15410 or NCIMB 14988 is twice more as much compared to that from strain WA or BC1, which is probably caused by their evolutionary relationships (Fig. 1). Microbial strains from the *Clostridium* genus have a wide and efficient utilization of a variety of carbon sources such as glucose, galactose, xylan and other polysaccharides. However, as indicated in Table 2, there are many significant differences among these five strains, especially in strains WA and WB. It was observed that strain WA is able to participate in the metabolic pathways involved not only in lactose, trehalose utilization and pyruvate conversion into butanoate, but also in the sulfoquinovosyl diacylglycerol biosynthesis, superoxide radicals degradation and thioredoxin pathway, whereas the number of metabolic pathways in strain WB was observed to be much higher than that in other strains. In addition, strains WA and BC1 had specific pathways involved in the biosynthesis of cellulose, fatty acids and 2,3-dihydroxybenzoate, whereas the metabolic pathways of gluconeogenesis and CDP-diacylglycerol biosynthesis were exclusively observed in strain WB, DSM 15410 and NCIMB 14988. These findings indicate the strong correlation between functional evolutionary relationships of five strains and their metabolic pathways.

In addition, five Clostridial strains can participate in a series of energy metabolism including biofuels production, nitrogen fixation, iron-sulfur cluster biosynthesis, etc. (Table 2). The energy metabolism involved in the sulfoquinovosyl diacylglycerol biosynthesis and the thioredoxin pathway were only discovered in strain WA; however, no such pathways were observed in strain WB, similar to that found in strain DSM 15410 or NCIMB 14988. Moreover, strain WA and BC1 have PTS-Dgl-gamP (d-glucosamine specific PTS), whereas PTS-Mal-malX (maltose specific PTS) is only involved in strain WB, DSM 15410 and NCIMB 14988. There is no doubt that all of the microorganisms, including *Clostridium* species, should possess vital metabolic pathways in the membrane transport, signal transduction and signaling molecules and interaction to adapt and respond to the culture environments; thus, the comparative genomic analysis of strains WA and WB with the other Clostridial species offers a better understanding of the substrate utilization process and bio-products generation [48].

### Reconstruction of the biofuels-related metabolic pathway via Clostridial genomes

**Fig. 5:**
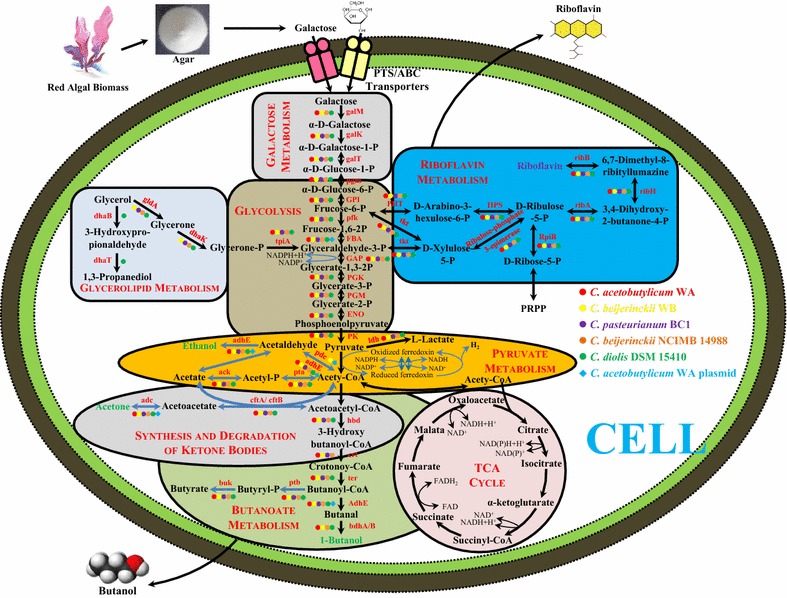
Reconstruction of the metabolic pathways involved in utilizing red algal biomass for generating various bio-products by *Clostridium* species. Symbols with color indicate different Clostridial strains or plasmids

So far, studies on the mechanism and regulation of sugar uptake and transport still remain limited; therefore, the newly isolated strains WA and WB were used to evaluate the feasibility of biofuels (e.g., biobutanol and biohydrogen) generation from galactose, the main component from red algal biomass [49, 50]. Via the reconstruction of the biofuels/biochemicals-related metabolic pathways of strains WA and WB with the references of three other representative Clostridial strains (Fig. 5), the obvious differences between the genomes of strains WA and WB are demonstrated in Table 5. It was observed that the genes of *galM* (aldose-1-epimerase), *galK* (galactokinase) and *galT* (galactose-1-phosphate uridylyltransferase) within the Leloir (LP) pathway for the conversion of galactose into α-d-glucose-1-P were crucial in both strains WA and WB [33]. Moreover, the number of genes participating in the carbohydrate metabolism from strain WB was also found to be higher than that from WA (Table 5), such as phosphoglucomutase (pgm) (Fig. 5).

In addition, *C. pasteurianum* BC1 was also reported to produce various biofuels (e.g., biobutanol and biohydrogen) [51, 52], and *C. diolis* DSM 15410 was identified as a 1,3-propanediol (1,3-PDO) producer [53]; however, *C. beijerinckii* NCIMB 14988 was used as a common strain for the evolutionary analysis without available bio-products shown [54]. Therefore, the metabolic pathways involved in the production of butanol, hydrogen and 1,3-PDO, together with another potential product (riboflavin, VB_2_), were reconstructed to better elaborate the possible generation of bio-products by strain WA and WB (Fig. 5). Same as strains NCIMB 14988, DSM 15410 and BC1, both strains WA and WB also possess all the crucial genes to complete the biosynthesis of riboflavin (Fig. 5, Table 2), which indicated the potential of these strains to produce riboflavin directly from galactose when an optimal cultivating condition is provided. Zhao et al. [55] reported that *C. acetobutylicum* ATCC 824 could generate riboflavin as a by-product during its ABE fermentation process via the supplementation of sodium acetate, and they also mentioned that the synthetic rate of GTP (precursor of riboflavin) could lead to the generation of riboflavin. Through the over-expression of riboflavin biosynthesis-related operon genes (*ribGBAH*) from strain ATCC 824, 20 mg/L of riboflavin could be determined extracellularly from *E. coli* [56]. Therefore, with the identification of the riboflavin metabolic pathway from strains WA and WB, it should be emphasized that the occurrence of the complete RBP (riboflavin biosynthetic pathway) system in both strains WA and WB would improve their possibility of producing riboflavin by regulating various external factors, such as culture pH, precursors or transporters [55, 57]. In total, the findings from the reconstruction of the powerful and complicated metabolic systems in Clostridial strains will facilitate the exploration of possible carbohydrate utilization and value-added products generation via the biochemical and/or molecular methodology [5].

### Determination of biofuels production by galactose-utilizing strains WA and WB

**Fig. 6:**
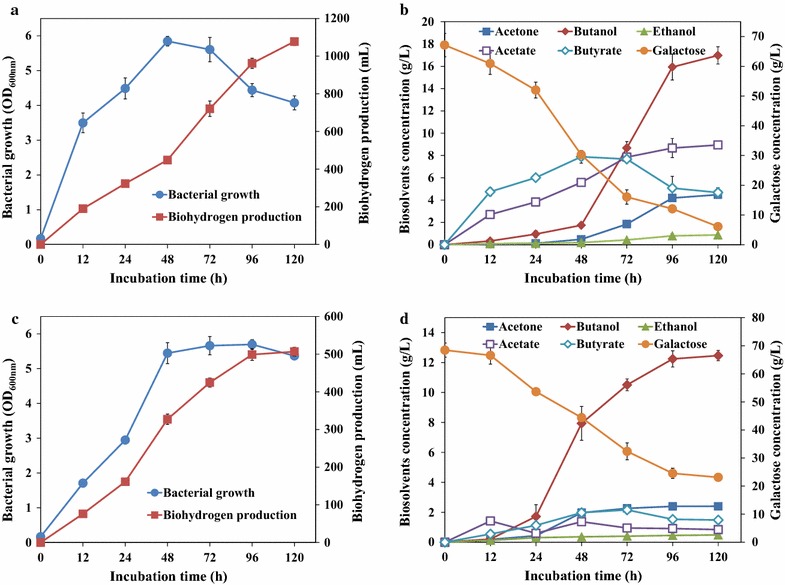
The anaerobic fermentation process of strain WA (**a**, **b**) and strain WB (**c**, **d**) using galactose (60 g/L) as the substrate

To verify the potential of biofuels production from galactose, a batch fermentation process was conducted using strains WA and WB in the defined culture medium [58] supplemented with 60 g/L of galactose, respectively. As shown in Fig. 6, strain WA produced 0.95 g/L butanol, 0.11 g/L ethanol and 190 mL hydrogen at the acidogenic stage with the decrement of pH value from 6.5 to 4.0 and relatively fast bacterial growth (OD_600 nm_ = 3.5) during the first 24 h (Fig. 6a). Followed by a second solventogenic stage, strain WA can produce up to 16.98 g/L butanol, 0.88 g/L ethanol and 1077.67 mL hydrogen by consuming almost all of the substrates after 120 h of fermentation (Fig. 6b). Strain WB generated a higher amount of butanol (1.72 g/L) with less acidic intermediates in the first 24 h (Fig. 6c), and finally produced 12.47 g/L of butanol with a very limited amount of ethanol (0.47 g/L) and 506.67 mL hydrogen after 120 h of fermentation (Fig. 6d). The yields of biohydrogen (353.21 and 165.99 mL/g) and biobutanol (0.283 and 0.207 g/g) from WA and WB, respectively, appeared to be much higher than that from other reported butanol-producing bacterial strains (Tables 3 and 4), which indicated a high possibility for adopting these two strains for bioconversion of biofuels from those galactose-rich substrates such as biomass from red alga. However, there were some obvious differences observed between these two strains, such as (i) the rate of galactose utilization by strain WA was much higher rather than that by strain WB (90.93% versus 66.20%), which led to a high production of butanol and hydrogen (Fig. 6a, c); (ii) a neglectable amount of ethanol was detected during the fermentation by strain WB, which was highly related to the lack of the pyruvate decarboxylase (*pdc*) gene within the genome of strain WB (Fig. 5, Table 5); and (iii) more butanol was formed from strain WA rather than that from strain WB during the fermentation, probably due to the lower amount of butanol synthesis-related gene (*bdhB*) in the genome of strain WB (Table 5). The butanol dehydrogenases A/B (*bdhA/B*), which are the critical enzymes for biobutanol synthesis, are both NADH dependent. However, the generation of butyrate during the ABE fermentation could consume ATP and inhibit the synthesis of NADH, which would further affect butanol yield [59, 60], and the pH adjustment during the fermentation could inhibit butyrate kinase (*buk*) to effectively reduce this influence [61]. With the occurrence of both *bdhA* and *bdhB* genes in strain WA, a significantly high amount of butanol was observed from the fermentation of strain WA with the pH adjustment (Fig. 6).

**Table 3:** Comparison of biohydrogen production of strains WA and WB with other reported *Clostridium* species

Bacterial strains	Substrate	Production (mL/L)	Yield (mL/g)^a^	References
*C. butyricum* CGS5	Xylose	–	108.17	[73]
*C. beijerinckii* DSM791	Glycerol	2682	292.60	[74]
*C. pasteurianum* CH4	Glycerol	–	268.41	[75]
*C. beijerinckii* IB4	Glucose	8240	137.33	[76]
*C. butyricum* CGS5	Sucrose	3577	180.68	[77]
*C. pasteurianum* MTCC116	Glycerol	–	210.38	[78]
*C. acetobutylicum* WA	Galactose	21,560	353.17	This study
*C. beijerinckii* WB	Galactose	10,140	165.47	This study

“–” data not available

^a^Values were calculated based on the consumed concentration of substrates

**Table 4:** Comparison of the biobutanol production of strains WA and WB with other reported *Clostridium* species

Bacterial strains	Substrate	Production (g/L)	Yield (g/g)^a^	References
*C. tyrobutyricum* Ct(Δack)-pscrBAK	Sucrose	16.0	0.31	[79]
*C. beijerinckii* IB4	Glucose	12.06	0.23	[76]
*C. pasteurianum* GL11	Glucose	5.0	0.08	[80]
*C. pasteurianum* GL11	Glycerol	14.7	0.25	[80]
*C. pasteurianum* ATCC6103	Glycerol	10.0	0.11	[81]
*C. pasteurianum* MBEL_GLY2	Glycerol	17.8	0.22	[81]
*C. acetobutylicum* WA	Galactose	16.98	0.28	This study
*C. beijerinckii* WB	Galactose	12.47	0.21	This study

^a^Values were calculated based on the consumed concentration of substrates

**Table 5:** Comparison of relevant genes involved in the butanol production by utilizing galactose between *Clostridium* sp. strain WA and WB

Genes involved	Genes counts	
	WA Chr (pWA)	WB Chr
Aldose 1-epimerase (*galM*)	1 (0)	3
Galactokinase (*galK*)	1 (0)	1
Galactose-1-phosphate uridylyltransferase (*galT*)	2 (0)	2
Phosphoglucomutase (*pgm*)	2 (0)	4
Glucose-6-phosphate isomerase	1 (0)	1
6-Phosphofructokinase (*pfk*)	1 (0)	3
Fructose-bisphosphate aldolase (*FBA*)	1 (1)	3
Glyceraldehyde-3-phosphate dehydrogenase (*GAP*)	2 (0)	1
Phosphoglycerate kinase (*PGK*)	1 (0)	1
Pyruvate decarboxylase (*pdc*)	0 (1)	0
Acetaldehyde dehydrogenase/alcohol dehydrogenase	1 (2)	2
NAD-dependent 4-hydroxybutyrate dehydrogenase (*hbd*)	2 (0)	2
3-Hydroxybutyryl-CoA dehydratase (*crt*)	2 (0)	2
NADH-dependent butanol dehydrogenase A (*bdhA*)	1 (0)	1
NADH-dependent butanol dehydrogenase B (*bdhB*)	1 (0)	0

*Chr* chromosomal DNA, *pWA* the plasmid DNA of strain WA

In this study, the genomes of two newly isolated Clostridial strains, WA and WB, with efficient galactose utilization were analyzed to illustrate their capability of synthesizing biofuels and/or biochemicals production, which might be improved via a future gene-engineering process without changing the native functional features. The genomic comparisons with other typical *Clostridium* species involved in various metabolic pathways also facilitate extension of the current limited understanding of their potential capability of using marine algal biomass as a sustainable substrate. In addition, CRISPR/Cas9-based genome editing using the plasmid of pNICKclos was recently developed to achieve an editing efficiency up to 100% in both *C. acetobutylicum* ATCC 824 and *C. beijerinckii* NCIMB 8052, two Clostridial strains with a complete genomic interpretation [62]. Therefore, with a more in-depth elaboration, strains WA and WB will demonstrate their demands of genome editing and regulation control, which also accelerates the progress in further metabolic engineering of the solventogenic Clostridia, regardless of the utilization of sustainable substrates or the generation of potentially industrial products.

## Conclusions

Via the comprehensive study of phylogeny and the genomic comparisons for two galactose-utilizing *Clostridium* strains identified to be *C. acetobutylicum* strain WA and *C. beijerinckii* strain WB, we provide a useful approach to highlight significant differences in biofuels-related metabolism. Furthermore, the results also demonstrate that potential products, such as riboflavin, were identified in the *Clostridium* metabolic pathway with marine biomass. Finally, the present work further extends our current understanding of Clostridia and provides a systematic investigation into the relationship between this genus and the generation of sustainable bioenergy.

## Methods

### Bacterial strains and cultivation conditions

The bacterial strains WA and WB with the capability of utilizing galactose as the sole carbon source were both isolated from mangrove sediments via enrichment using the reinforced clostridial medium (RCM). The cultivation medium of these two strains was prepared by using the defined culture medium amended with 60 g/L of galactose as described by Wu et al. [63].

### Genome sequencing and re-annotation of strains WA and WB

The genomic DNA of strains WA and WB was extracted using the E.Z.N.A.^®^ Bacterial DNA Kit (Omega Bio-Tek, USA) according to manufacturers’ instructions, and applied to whole shotgun sequencing using the Illumina paired-end sequencing technology at the Beijing Genomics Institute (BGI, China). The obtained reads were assembled into contigs in different scales by using SOAPdenovo (V1.05). The re-annotation of whole genomes, including the functional genes and RNA prediction, was performed using the prokaryotic genome annotation system pipeline program (V1.11) [64], and the identification and classification of the encoded functional proteins was determined based on the Clusters of Orthologous Groups (COG) database.

### Whole genome-based phylogenetic analysis of Clostridial strains

Genomes from strains WA and WB, together with those from 33 other bacterial strains from the *Clostridium* genus and an outgroup strain (*Bacillus licheniformis* ATCC 14580) affiliated to the same *Clostridiaceae* family were analyzed to establish their phylogenetic relationship. By referring to the method from Sun et al. [65], a Clostridial phylogenetic tree was finally constructed with the LG substitution matrix and the gamma model using the RAxML tool (V8.0) based on a concatenation of 10793 amino acid sites over 129 single-copy gene families shared by 36 available genomes [38] and finally demonstrated via MEGA6 [66].

### Comparative genomic analysis on the genome-wide metabolic pathway

To verify the differential genome-wide metabolic pathways among the Clostridial strains, *C. acetobutylicum* strain WA and *C. beijerinckii* strain WB together with their phylogenetically close strains, *C. beijerinckii* NCIMB 14988, *C. pasteurianum* BC1 and *C. diolis* DSM 15410, were selected as the representative strains for genomic comparison. Circos [67] and Mauve [68] software was used to compare the assembly differences. The MetaPathways software (V2.0) was further adopted to re-construct the genome-wide metabolic pathways with the following parameters: (i) ORFs detection by Prodigal with minimal length of 60 amino acids and (ii) functional annotation via BLAST with an *e* value of 10^−5^ and a Blast-score ratio of 0.4 [69, 70] using the protein databases of KEGG, CAZY, COG, MetaCyc and RefSeq. In addition, the metabolic pathways involved in algal biomass utilization and biochemicals/biofuels synthesis were further validated through the databases of KEGG and TCDB, and reconstructed using Adobe Illustrator CS6 software.

### Determination of fermentative products by strain WA and WB

To determine both strains WA and WB and their potential for fermentative products generation using galactose, the activated strains WA and WB were inoculated into 50 mL of galactose-supplemented culture medium and cultivated at 37 °C in a shaker with a rotary speed of 150 rpm for 96 h, respectively. The pH of the culture medium was manually maintained at 5.0–5.5 using a 3 M NaOH solution during the entire fermentation process. Starting from 0 h of fermentation, the production of biohydrogen and biosolvents (i.e., acetone, butanol, ethanol, acetic acid and butyric acid), the bacterial growth as well as the substrate consumption were recorded every 12 or 24 h. Hydrogen was collected by gas sampling bags and determined using a GC-2010Plus gas chromatograph (GC) equipped with a thermal conductivity detector (TCD) and a Supelco 80/100, Porapak-N column (Shimadzu, Japan) as described by the method of Wu et al. [58]. The oven temperature was maintained at 110 °C for 5 min, and argon was used as the carrier gas with a column flow rate of 1 mL/min. Standard gaseous mixtures, which consists of nitrogen (60%), carbon dioxide (15%), carbon monoxide (15%), hydrogen (5%) and methane (5%), were used for quantification. The concentration of the biosolvents was also measured by a GC-2010Plus GC equipped with a flame ionization detector (FID) and a DB-WAXetr column (30 m × 0.25 mm × 0.25 μm ID) based on the method of Xin et al. [71] with minor modifications. The oven temperature was initially held at 60 °C for 2 min, increased at 15 °C/min to 230 °C, and was then held for 1 min. Helium was used as the carrier gas with a column flow rate of 1 mL/min and a mixed biosolvents standard curve was established for quantification. The bacterial biomass was assessed using a UVmini-1240 UV–visible spectrophotometer (Shimadzu, Japan) at a wavelength of 600 nm, whereas the concentration of galactose was measured using the 3,5-dinitrosalicylic acid (DNS) method [72].

## Additional file

**Additional file 1: **The genomic characteristics of the representative Clostridial strains. **Table S1.** The genomic characteristics of 35 *Clostridium* strains (without plasmids). **Table S2.** The characteristics of 8 plasmids.

**Additional file 2: Figure S1.** A comparative genomic analysis between strain WA and WB using Mauve software. A: Chromosomal DNA of strain WA; B: Chromosomal DNA of strain WB; and C: Plasmid DNA of strain WA.
