# Supplementary material for: Genomic comparison of Clostridium species with the potential of utilizing red algal biomass for biobutanol production
Source: Biotechnol Biofuels. 2018 Feb 15;11:42. doi: 10.1186/s13068-018-1044-9 (PMC5815214; doi:10.1186/s13068-018-1044-9)
Supplement: Supplementary file 1 — Additional file 1: The genomic characteristics of the representative Clostridial strains. Table S1. The genomic characteristics of 35 Clostridium strains (without plasmids). Table S2. The characteristics of 8 plasmids. [file 13068_2018_1044_MOESM1_ESM.docx]

**Table S1 The genomic characteristics of 35 *Clostridium* strains (without plasmids).**

| **Strain No.** | **Strain names** | **Size (Mb)** | **GC(%)** | **Genes** | **CDSs** | **rRNAs** | **tRNAs** | **Other RNAs** |
| --- | --- | --- | --- | --- | --- | --- | --- | --- |
| 1 | *C. aceticum* DSM 1496 | 4.2 | 35.3 | 3,847 | 3,705 | 18 | 74 | 1 |
| 2 | *C. acetireducens* DSM 10703 | 2.42 | 26.7 | 2,301 | 2,162 | 26 | 69 | 5 |
| **3** | ***C. acetobutylicum* WA** | **4.07** | **30.8** | **3,927** | **3,878** | **31** | **72** | **1** |
| 4 | *C. amylolyticum* DSM 21864 | 4.26 | 32.1 | 4,050 | 3,900 | 10 | 70 | 4 |
| 5 | *C. arbusti* SL206 | 3.97 | 29.8 | 3,633 | 3,417 | 10 | 70 | 1 |
| 6 | *C. argentinense* CDC 2741 | 4.74 | 28.6 | 4,214 | 4,027 | 19 | 59 | 4 |
| 7 | *C. autoethanogenum* DSM 10061 | 4.35 | 31.1 | 3,983 | 3,741 | 27 | 67 | 1 |
| 8 | *C. baratii* str. Sullivan | 3.15 | 28.3 | 2,999 | 2,871 | 31 | 78 | 1 |
| 9 | *C. beijerinckii* NCIMB 14988 | 6.49 | 29.6 | 5,845 | 5,701 | 49 | 94 | 6 |
| **10** | ***C. beijerinckii* WB** | **5.78** | **29.7** | **5,142** | **5,085** | **4** | **56** | **1** |
| 11 | *C. butyricum* KNU-L09 | 3.82 | 28.8 | 3,394 | 3,216 | 33 | 87 | 1 |
| 12 | *C. cadaveris* AGR2141 | 3.54 | 31.1 | 3,378 | 3,214 | 20 | 53 | 1 |
| 13 | *C. cavendishii* DSM 21758 | 4.99 | 27.9 | 4,529 | 4,315 | 19 | 63 | 4 |
| 14 | *C. cellulovorans* 743B | 5.26 | 31.2 | 4,384 | 4,230 | 28 | 80 | 2 |
| 15 | *C. collagenovorans* DSM 3089 | 3.48 | 28.9 | 3,046 | 2,921 | 18 | 49 | 4 |
| 16 | *C. cylindrosporum* DSM 605 | 2.74 | 31.4 | 2,650 | 2,535 | 9 | 65 | 1 |
| 17 | *C. diolis* DSM 15410 | 5.85 | 29.7 | 5,147 | 4,914 | 26 | 74 | 6 |
| 18 | *C. fallax* DSM 2631 | 2.75 | 26.5 | 2,575 | 2,426 | 12 | 84 | 4 |
| 19 | *C. grantii* DSM 8605 | 4.47 | 29.3 | 4,234 | 3,984 | 21 | 52 | 5 |
| 20 | *C. homopropionicum* DSM 5847 | 3.65 | 31.1 | 3,544 | 3,376 | 30 | 76 | 1 |
| 21 | *C. ihumii* AP5 | 4.43 | 26.7 | 4,075 | 3,897 | 13 | 75 | 4 |
| 22 | *C. kluyveri* DSM 555 | 3.96 | 32 | 3,824 | 3,712 | 20 | 60 | 1 |
| 23 | *C. novyi* NT | 2.55 | 28.9 | 2,381 | 2,237 | 30 | 81 | 2 |
| 24 | *C. paraputrificum* AGR2156 | 3.56 | 29.6 | 3,458 | 3,345 | 18 | 46 | 1 |
| 25 | *C. pasteurianum* BC1 | 4.99 | 30.6 | 4,643 | 4,420 | 27 | 74 | 8 |
| 26 | *C. perfringens* ATCC 13124 | 3.26 | 28.4 | 2,932 | 2,782 | 24 | 92 | 2 |
| 27 | *C. phoceensis* GD3 | 6.9 | 59.3 | 3,432 | 3,236 | 5 | 51 | 4 |
| 28 | *C. ragsdalei* P11 | 4.42 | 31 | 4,004 | 3,824 | 32 | 68 | 6 |
| 29 | *C. saccharoperbutylacetonicum* N1-4(HMT) | 6.53 | 29.5 | 5,678 | 5,543 | 35 | 70 | 1 |
| 30 | *C. sartagoforme* AAU1 | 3.98 | 27.9 | 3,878 | 3,478 | 8 | 64 | 1 |
| 31 | *C. tepidiprofundi* DSM 19306 | 3.06 | 29.4 | 2,856 | 2,598 | 24 | 91 | 5 |
| 32 | *C. tetani* E88 | 2.8 | 28.7 | 2,722 | 2,620 | 18 | 54 | 1 |
| 33 | *C. tunisiense* TJ | 4.31 | 31.2 | 3,956 | 3,766 | 19 | 104 | 1 |
| 34 | *C. tyrobutyricum* KCTC 5387 | 3.07 | 31.1 | 3,005 | 2,877 | 19 | 63 | 5 |
| 35 | *C. akagii* DSM 12554 | 4.59 | 30.7 | 4,313 | 4,112 | 15 | 67 | 1 |

**Table S2 The characteristics of 8 plasmids.**

| **Plasmid No.** | **Strain names** | **Plasmid names** | **Size (Mbp)** | **GC%** | **CDSs/Genes** |
| --- | --- | --- | --- | --- | --- |
| 1 | *C. aceticum* DSM 1496 | CACET_5p | 0.005719 | 31.7 | 8 |
| 2 | *C. acetobutylicum* WA | pWA | 0.191 | 30.8 | 178 |
| 3 | *C. baratii* str. Sullivan | pCBJ | 0.185364 | 27.6 | 216 |
| 4 | *C. kluyveri* DSM 555 | pCKL555A | 0.059182 | 33.4 | 79 |
| 5 | *C. pasteurianum* BC1 | pCLOPA01 | 0.053393 | 28.9 | 56 |
| 6 | *C. saccharoperbutylacetonicum* N1-4(HMT) | Csp_135p | 0.136188 | 29.4 | 106 |
| 7 | *C. tetani* E88 | pE88 | 0.074082 | 24.5 | 97 |
| 8 | *C. tyrobutyricum* KCTC 5387 | pCTK01 | 0.062831 | 27.6 | 71 |
