# Supplementary material for: Genomic comparison of Clostridium species with the potential of utilizing red algal biomass for biobutanol production
Source: Biotechnol Biofuels. 2018 Feb 15;11:42. doi: 10.1186/s13068-018-1044-9 (PMC5815214; doi:10.1186/s13068-018-1044-9)
Supplement: Supplementary file 2 — Additional file 2: Figure S1. A comparative genomic analysis between strain WA and WB using Mauve software. A: Chromosomal DNA of strain WA; B: Chromosomal DNA of strain WB; and C: Plasmid DNA of strain WA. [file 13068_2018_1044_MOESM2_ESM.docx]

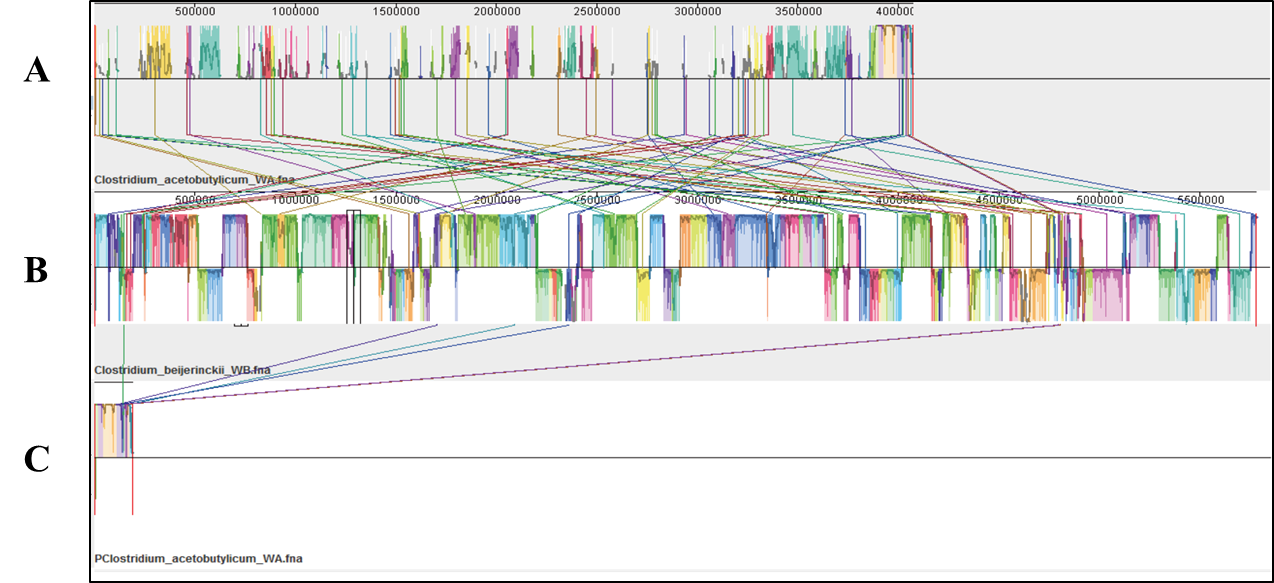


**Fig. S1 A comparative genomic analysis between strain WA and WB using Mauve software.** A: Chromosomal DNA of strain WA; B: Chromosomal DNA of strain WB; and C: Plasmid DNA of strain WA.
